# Paternal Age and General Cognitive Ability—A Cross Sectional Study of Danish Male Conscripts

**DOI:** 10.1371/journal.pone.0077444

**Published:** 2013-10-08

**Authors:** John McGrath, Preben Bo Mortensen, Carsten Bøcker Pedersen, Vera Ehrenstein, Liselotte Petersen

**Affiliations:** 1 Queensland Brain Institute, The University of Queensland, St. Lucia, Australia; 2 Queensland Centre for Mental Health Research, The Park Centre for Mental Health, Richlands, Australia; 3 National Centre for Register-Based Research, Aarhus University, Aarhus, Denmark; 4 The Lundbeck Foundation Initiative for Integrative Psychiatric Research, iPSYCH, Aarhus, Denmark; 5 CIRRAU, Aarhus University, Aarhus, Denmark; 6 Department of Clinical Epidemiology, Aarhus University Hospital, Aarhus, Denmark; Cardiff University, United Kingdom

## Abstract

**Objectives:**

Offspring of older men have impaired cognitive ability as children, but it is unclear if this impairment persists into adulthood. The main objective of this study was to explore the association between paternal age at offspring birth and general cognitive ability as young adults.

**Design:**

Population-based cross-sectional study with prospectively collected data on obstetric factors and parental education.

**Setting:**

Nationwide Danish sample.

**Participants:**

Male conscripts (n = 169,009).

**Primary and secondary outcome measures:**

General cognitive ability as assessed by the Børge Priens test score, an intelligence test with components related to logical, verbal, numerical and spatial reasoning.

**Results:**

We observed an inverse U-shaped association between paternal age and general cognitive ability (slightly lower test scores in the offspring of fathers aged less than 25 years and older than 40 years, compared with fathers aged 25 to 29 years). However, after adjustment for maternal age, parental education and birth order the shape of the association changed. Offspring of fathers younger than 20 still showed slightly lower cognitive ability (-1.11 (95% CI -1.68 to -0.54)), but no significant impairments were identified in the men whose fathers were older than 29 years at the time of their birth (e.g. the mean difference in test score in the offspring of fathers aged 40 to 44 years were -0.03 [95% CI (-0.27 to 0.20)] compared with fathers aged 25 to 29 years).

**Conclusions:**

We did not find that the offspring of older fathers had impaired cognitive ability as young adults. Whereas, we found a tendency that the offspring of teen fathers have lower cognitive ability. Thus, our results suggest that any potentially deleterious effects of older fathers on general cognitive ability as young adults may be counter-balanced by other potentially beneficial factors.

## Introduction

In recent years there has been renewed interest in the links between advanced paternal age and adverse health outcomes in the offspring [1-8]. Recently published genetic studies have demonstrated that the offspring of older fathers have more *de novo* mutations, which is thought to be related to the greater number of cell divisions in the male versus female germ line [9-14]. Apart from the association between paternal age at the time of child’s birth and risk of neurodevelopmental disorders such as schizophrenia and autism [15], there is a small body of literature focussed on cognitive and educational outcomes with respect to paternal age. The results of these studies have been mixed. For example, a study among Israeli conscripts (male and female, age 16-17 years, n = 44,175) found an inverse U-shaped association between paternal age and intelligence scores [16]. Studies based on a large US birth cohort (samples 20,000 to 30,000) suggested that offspring of older fathers have impaired performance on various neurocognitive measures at age 8 months, 4 and 7 years [17], however, this association was attenuated by adjustment for sibship size [18].

Recently, two Swedish studies have provided additional evidence suggesting that the pattern of relationship between paternal age and cognitive ability may be more complicated. Svensson and colleagues examined a composite educational score among children in Stockholm who completed 9 years of compulsory schooling (n = 155,875) - they found that children of older fathers did not have worse school performance [19]. Another, larger Swedish study, based on male conscripts (n = 565,433) examined the association between paternal age and a measure of general intelligence, assessed between age 17 and 20 years [20]. This study found that the offspring of both young and older fathers had lower scores on the intelligence measure compared with the offspring of fathers aged 25-29 years. When comparing brothers, and adjusting for potential confounding variables (birth order, age at testing, conscription centre, year of testing) the effect of paternal age on intelligence was attenuated. Studies have also reported that the association between maternal age and cognitive outcomes in young adults is an inverted U-shape [16,20], whereas the studies that have examined neurocognitive measures in childhood report that the children of older mothers tend to perform better [17,18].

Apart from paternal age-related mutations, complex mechanisms could underlie the association between paternal age at the time of birth and cognitive function in offspring. For example, those with higher intelligence are more likely to undertake university education, and because intelligence is heritable [21], selective factors may operate (i.e. parents with higher education may reproduce later in life) [22]. Furthermore, paternal age correlates with birth order, while higher birth order is generally associated with poorer cognitive outcomes (see reviews [23,24]). If larger family size is associated with lower socio-economic status, this factor can confound the association between paternal age, birth order and cognitive function [23]. Curiously, while first born offspring (who tend to have younger fathers) do better than later born offspring on intelligence measures when tested before the age of 12 years, this effect can reverse when the offspring are assessed after age 12 years [25]. Birth weight is associated with IQ [26] and is correlated to paternal age, therefore we wished to explore if paternal age influenced cognitive scores when the influence of birth weight and gestational age were controlled for. In summary, there appears to be biological and psychosocial factors influencing the association between paternal age and offspring cognitive outcomes across childhood and young adulthood.

We examined the association between paternal age and cognitive ability in young men in a large Danish record linkage study. In particular, we were interested in how a broad range of variables may influence the association between paternal age and general cognitive ability. Because many of the variables are (a) highly intercorrleted (e.g. paternal age versus maternal age, birth order versus paternal age), (b) potentially operate as confounds (e.g. greater paternal education could serve both to delay paternal age and influence the intelligence of the offspring via a range of mechanisms) and (c) potentially mediate the association between paternal age and cognitive ability. Where variables are highly intercorrelated, and if we have an incomplete understanding of underlying causality within the matrix of variables, making adjustments for potentially mediating variables may have unintended consequences (e.g. overadjustment, spurious associations) [27-29]. In order to aid the research community explore the influence of a range of variables that could link paternal age and cognitive ability, we will show a range of models that adjust for a broad range of variables and examine the risk sets that reduce the influence of potential mediators (e.g. gestation, birth weight, singleton status). Based on the evidence linking advanced paternal age and increased risk of neurodevelopmental outcomes such as autism and schizophrenia, we hypothesized that the offspring of older fathers would have lower scores on the intelligence-related measure in early adulthood.

## Methods

### Ethics approval

Approval was provided by the Danish Data Protection Agency. The study was based solely on national and administrative registers and did not require any approval from the ethics committee according to national regulations.

### Study population

This study was based on two independent samples of young Danish men: (a) a sample from draft board examinations in North Jutland, which included approx. 36,000 men born in Denmark between 1955 and 1984 and assessed between 1974 and 2002, and (b) the Danish Conscription Registry [30], a nationwide register, which included approx. 135,000 men born in Denmark between 1976 and 1993 and assessed between 2006 and 2010. The pooled data included 171,194 Danish men born 1955 to 1993. Men with conditions such as severe mental retardation, asthma and extreme myopia are exempted from conscription (approx 10-15%), but not all mental health problems are regarded as disqualifiers for military service [31,32]. All Danish residents are assigned a unique personal identifier in all Danish national registers, enabling unique linkage between registers. From the Danish Civil Registration System [33] information on parental age, birth order and twin status were obtained. The Danish Medical Birth Registry [34] contains maternal and obstetric information, such as gestational age and birth weight. From Statistics Denmark’s database IDA we have information on parental education [35]. We excluded 2094 conscripts with unknown fathers, 64 with unknown mothers, 27 with missing information on birth order or singleton versus multiple birth status, accordingly the analyses includes 169,009 conscripts.

### Outcome measure

The Børge Priens Prøve (BPP) is a Danish intelligence test has been used by the Danish Draft Board examination since 1956. The test consists of 4 subtests each with about 20 items (78 in total), designed to assess logical, verbal, numerical and spatial reasoning. The tests are timed and the result is number of correct answers to the 78 questions. The test has satisfactory test-retest reliability, and correlates with educational achievement and with the Wechsler Adult Intelligence Scale (correlation = 0.82) [36,37]. Mean values of the BPP test have changed over recent decades, thus it is recommended that studies account for year of testing [31,38].

### Statistical analysis

We estimated mean differences in BPP test scores and likelihood ratio based 95% confidence intervals according to parental ages using linear regression in Stata 10 (Stata, College Station, Texas, United States). Year of testing was categorized in approximately equal sized groups: 1974-83, 1984-89, 1990-94, 1995-99, 2000-05, 2006-09. Parental ages at the time of the conscript’s birth were categorized. As the reference category, we used age group 25 to 29 years old at the time of the conscripts’ birth. Estimates in the initial models were all adjusted for birth year, birth weight was adjusted for gestational age and vice versa, likewise; paternal was adjusted for maternal age and vice versa. Estimates in the full model were based on term-born (gestational age of week 37 or later) conscripts while adjusting for paternal and maternal education, measured when the conscript was around 18 years of age, birth order, multiple birth status, birth weight and gestational age. To distinguish effects of restricting to term babies and the effect of adjustment an additional set of analyses were conducted; initial models in term babies. Our full model includes correlated variables and the causality from parental ages to BPP test scores are incompletely understood, thus we made a sequence of additional analyses. Estimated mean differences of BPP scores according to parental ages are presented unadjusted and for each of the other variables entered one at a time. Conscripts with the same father (i.e. brothers or paternal half-brothers) comprised a cluster, and allowance for possible within-cluster dependence was made by using robust standard error estimates provided by the cluster option in Stata.

## Results

**Figure 1:**
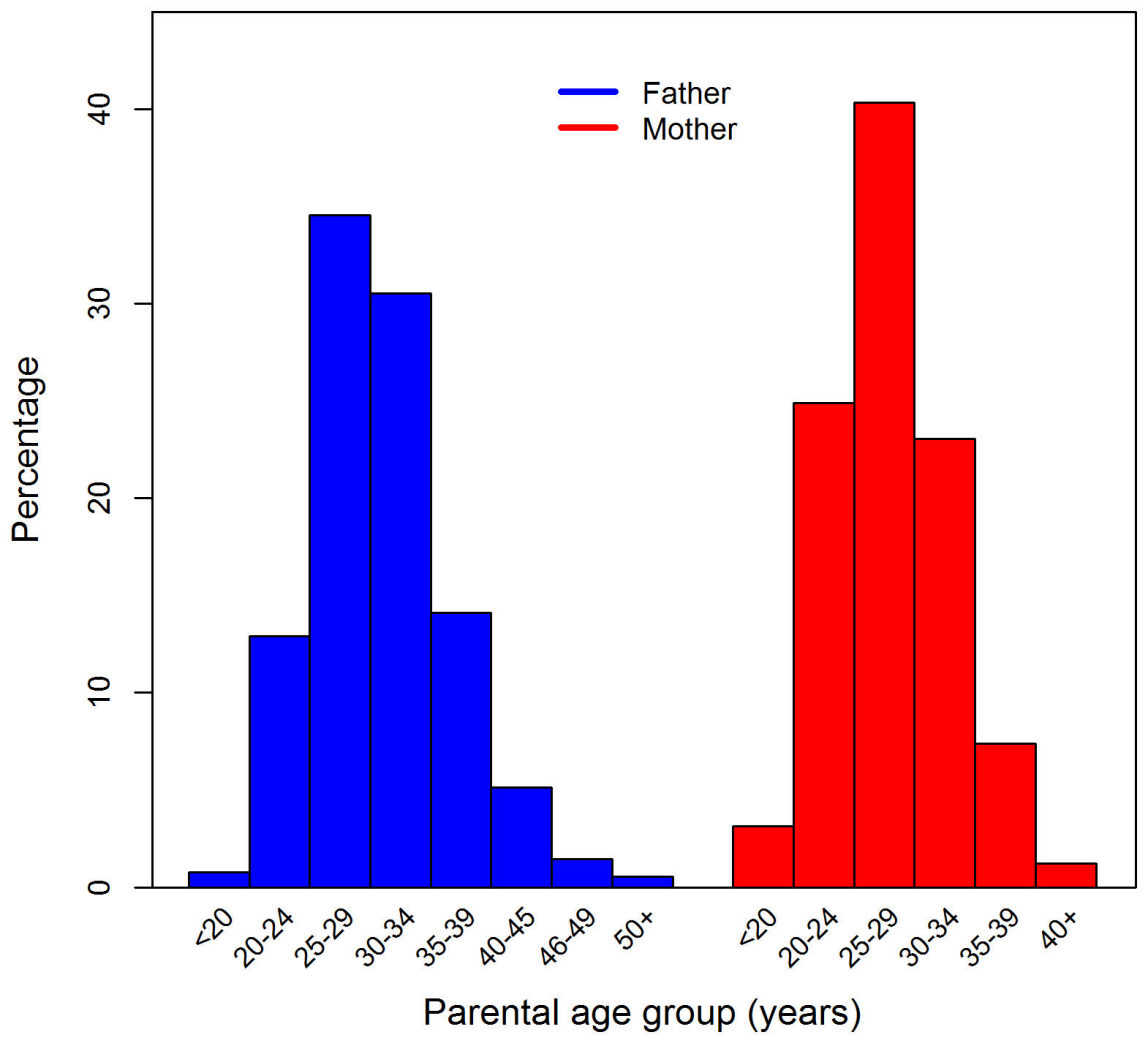
Distribution of parental age groups.

The mean (standard deviation) age of the 169,009 conscripts at testing was 19.4 (1.0) years. The overall mean and standard deviation of the BPP cognitive scores was 42.0 and 9.7 units (higher scores indicate better cognitive ability). The distribution of paternal and maternal age groups is shown in Figure 1. The analyses were initially conducted on the two samples independently, however, because the overall pattern of findings was similar, we pooled the samples for this analysis.

In the initial models we found an inverted U-shaped association between paternal age and the BP cognitive score, with both the offspring of younger and older fathers have lower mean scores compared with the reference category (see Table 1). For example, compared with the reference category, the offspring of father aged less than 20 years had a mean score of 1.62 units lower on the BP scores (95%CI -2.21 to -1.03), while the offspring of fathers 50 years or older had a mean score of 1.97 units lower (95%CI -2.68 to -1.26). Restricting the analyses to term babies did not change the estimates much, except for multiple pregnancy status. In the full model, the association between fathers as well as mothers younger than 20 years and lower BP scores persisted, whereas the offspring of older fathers tended to have higher scores. For example, the offspring of fathers aged 30-34 and 35-39 years old achieved slightly higher mean BP scores (0.75 (0.56 to 0.94) and 0.72 (0.50 to 0.95) higher BP scores, respectively).

**Table 1:** Mean differences in BPP test scores and 95% confidence intervals.

		Initial models*	Initial models in term babies**	Full model***
Paternal age (years)	Less than 20	-1.62 (-2.21 to -1.03)	-1.84 (-2.51 to -1.17)	-0.69 (-1.34 to -0.04)
	20-24	-0.83 (-0.99 to -0.66)	-0.87 (-1.05 to -0.69)	0.49 (0.32 to 0.66)
	25-29	0 (reference)	0 (reference)	0 (reference)
	30-34	0.17 (0.05 to 0.29)	0.21 (0.08 to 0.34)	0.75 (0.56 to 0.94)
	35-39	-0.16 (-0.33 to 0.01)	-0.07 (-0.25 to 0.12)	0.72 (0.50 to 0.95)
	40-44	-0.88 (-1.13 to -0.63)	-0.72 (-0.99 to -0.45)	0.50 (0.21 to 0.79)
	45-49	-1.24 (-1.66 to -0.81)	-1.00 (-1.46 to -0.55)	0.34 (-0.11 to 0.79)
	50 and above	-1.97 (-2.68 to -1.26)	-1.44 (-2.22 to -0.66)	0.36 (-0.39 to 1.10)
Maternal age (years)	Less than 20	-4.34 (-4.66 to -4.03)	-4.55 (-4.91 to -4.19)	-1.69 (-2.03 to -1.34)
	20-24	-1.88 (-2.01 to -1.75)	-1.92 (-2.06 to -1.78)	1.11 (0.97 to 1.24)
	25-29	0 (reference)	0 (reference)	0 (reference)
	30-34	0.70 (0.57 to 0.83)	0.77 (0.64 to 0.91)	1.66 (1.48 to 1.83)
	35-39	1.07 (0.87 to 1.29)	1.17 (0.94 to 1.39)	2.08 (1.83 to 2.33)
	40 and above	0.58 (0.11 to 1.05)	0.65 (0.12 to 1.18)	1.88 (1.36 to 2.40)
Paternal education	Basic	0 (reference)	0 (reference)	0 (reference)
	High school	2.58 (2.47 to 2.70)	2.53 (2.41 to 2.66)	1.72 (1.60 to 1.84)
	Vocational	6.00 (5.75 to 6.26)	6.00 (5.74 to 6.26)	4.01 (3.76 to 4.27)
	Short duration post-school	6.19 (6.05 to 6.33)	6.11 (5.96 to 6.25)	3.86 (3.70 to 4.01)
	Long duration post-school	9.05 (8.88 to 9.22)	8.98 (8.80 to 9.15)	5.36 (5.17 to 5.56)
Maternal education	Basic	0 (reference)	0 (reference)	0 (reference)
	High school	3.17 (3.05 to 3.29)	3.06 (2.94 to 3.19)	2.08 (1.95 to 2.21)
	Vocational	5.90 (5.67 to 6.13)	5.79 (5.55 to 6.03)	3.98 (3.74 to 4.22)
	Short duration post-school	6.75 (6.62 to 6.87)	6.66 (6.53 to 6.80)	4.14 (4.00 to 4.28)
	Long duration post-school	10.1 (9.85 to 10.3)	9.97 (9.76 to 10.2)	5.79 (5.55 to 6.03)
Birth order	1^st^ born	0 (reference)	0 (reference)	0 (reference)
	2^nd^ born	-0.69 (-0.79 to -0.59)	-0.72 (-0.82 to -0.61)	-1.40 (-1.51 to -1.30)
	3^rd^ born	-1.48 (-1.63 to -1.33)	-1.46 (-1.61 to -1.30)	-2.24 (-2.40 to -2.07)
	4^th^ or later born	-3.13 (-3.40 to -2.87)	-3.00 (-3.29 to -2.72)	-3.28 (-3.57 to -2.98)
Multiple pregnancy status	Singleton	0 (reference)	0 (reference)	0 (reference)
	Twin	-0.54 (-0.92 to -0.17)	-0.16 (-0.61 to 0.29)	0.61 (0.17 to 1.04)
	Triple or more	-2.15 (-4.61 to 0.30)	1.99 (-4.57 to 8.55)	2.08 (-2.36 to 6.51)
Birth weight (grams)	-2499	-2.06 (-2.39 to -1.73)	-2.39 (-2.80 to -1.97)	-2.09 (-2.49 to -1.70)
	2500-2999	-1.19 (-1.36 to -1.01)	-1.34 (-1.52 to -1.15)	-0.98 (-1.15 to -0.81)
	3000-3499	0 (reference)	0 (reference)	0 (reference)
	3500-3999	1.05 (0.93 to 1.17)	1.08 (0.96 to 1.20)	0.81 (0.69 to 0.92)
	4000-4499	1.67 (1.52 to 1.82)	1.72 (1.56 to 1.87)	1.36 (1.22 to 1.50)
	4500-	1.95 (1.68 to 2.23)	2.02 (1.74 to 2.30)	1.68 (1.42 to 1.94)
Gestational age	per week in preterm babies	0.01 (-0.08 to 0.11)	-	-
	per week in term babies	-0.08 (-0.12 to -0.04)	-0.07 (-0.11 to -0.03)	-0.10 (-0.14 to -0.06)

The association between parental ages and related variables versus BPP cognitive score at conscription (n = 169,009 in initial models, except that birth weight is available in 156,948).

* Initial models include year of testing, paternal and maternal ages are mutually adjusted, and birth weight and gestational age are mutually adjusted

** Term babies includes 145,780

*** In the full model all variables listed in the table were in the analysis

In the full model, higher BP cognitive score was associated with having an older mother; the BP score was in the range of 3.5 points higher in offspring of mothers aged 35 years or more compared to offspring of teen mothers. In addition, higher BP cognitive score was associated with better educated mother and father. The mean difference, e.g., between scores of offspring having a father with long duration education and a father with only basic schooling was 5.36 (5.17 to 5.56). Furthermore, higher BP cognitive score was associated with being first-born, gestational age close to 37 weeks, and higher birth weight.

In the crude model we found an inverted U-shaped association between paternal age and the BPP score, where the sons of fathers younger than 25 years have mean scores 2-4 units lower compared to the reference category, and sons of fathers older than 50 years had a mean score 1 unit lower compared to the reference category (see Table 2). When parental ages were mutually adjusted, both sons of young and old fathers have mean scores 1-2 units below the reference category, whereas mean scores tend to increase with increasing maternal age. Adjusting for parental education and birth order, the sons of older fathers was not significantly lower than the reference category. Restricting to term born singletons between 3 and 4 kilograms at birth does not change the overall pattern of associations.

**Table 2:** Mean differences in BPP test scores and 95% confidence intervals.

		Crude	Adjusted for year of testing	Parental ages mutually adjusted
Paternal age (years)	Less than 20	-4.28 (-4.84 to -3.73)	-4.21 (-4.77 to -3.66)	-1.62 (-2.21 to -1.03)
	20-24	-2.02 (-2.18 to -1.87)	-2.03 (-2.19 to -1.88)	-0.83 (-0.99 to -0.66)
	25-29	0 (reference)	0 (reference)	0 (reference)
	30-34	0.86 (0.75 to 0.98)	0.91 (0.80 to 1.02)	0.17 (0.05 to 0.29)
	35-39	0.84 (0.69 to 0.99)	0.96 (0.81 to 1.11)	-0.16 (-0.33 to 0.01)
	40-44	0.14 (-0.09 to 0.36)	0.35 (0.12 to 0.57)	-0.88 (-1.13 to -0.63)
	45-49	-0.39 (-0.79 to 0.02)	0.01 (-0.39 to 0.41)	-1.24 (-1.66 to -0.81)
	50 and above	-1.06 (-1.77 to -0.36)	-0.73 (-1.43 to -0.04)	-1.97 (-2.68 to -1.26)
Maternal age (years)	Less than 20	-5.05 (-5.33 to -4.76)	-5.04 (-5.33 to -4.75)	-4.34 (-4.66 to -4.03)
	20-24	-2.08 (-2.20 to -1.97)	-2.15 (-2.27 to -2.03)	-1.88 (-2.01 to -1.75)
	25-29	0 (reference)	0 (reference)	0 (reference)
	30-34	0.61 (0.49 to .73)	0.66 (0.54 to 0.77)	0.70 (0.57 to 0.83)
	35-39	0.58 (0.40 to 0.77)	0.72 (0.53 to 0.90)	1.07 (0.87 to 1.29)
	40 and above	-0.55 (-1.01 to -0.10)	-0.19 (-0.64 to 0.26)	0.58 (0.11 to 1.05)
		Adjusted for parental education	Adjusted for parental education and birth order	In singletons born week 37-41 and birth weight 3-4 kg
Paternal age (years)	Less than 20	-0.89 (-1.46 to -0.31)	-1.11 (-1.68 to -0.54)	-1.25 (-2.05 to -0.44)
	20-24	-0.37 (-0.53 to -0.21)	-0.52 (-0.68 to -0.36)	-0.46 (-0.67 to -0.25)
	25-29	0 (reference)	0 (reference)	0 (reference)
	30-34	0.08 (-0.03 to 0.20)	0.26 (0.15 to 0.38)	0.23 (0.08 to 0.39)
	35-39	-0.03 (-0.19 to 0.13)	0.24 (0.08 to 0.40)	0.34 (0.12 to 0.55)
	40-44	-0.26 (-0.50 to -0.03)	-0.03 (-0.27 to 0.20)	-0.03 (-0.35 to 0.29)
	45-49	-0.27 (-0.67 to 0.12)	-0.17 (-0.56 to 0.23)	-0.29 (-0.84 to 0.25)
	50 and above	-0.46 (-1.13 to 0.21)	-0.51 (-1.18 to 0.16)	-0.02 (-0.93 to 0.88)
Maternal age (years)	Less than 20	-2.20 (-2.51 to -1.89)	-2.85 (-3.16 to -2.54)	-2.87 (-3.32 to -2.43)
	20-24	-0.77 (-0.90 to -0.64)	-1.11 (-1.24 to -0.98)	-1.15 (-1.32 to -0.98)
	25-29	0 (reference)	0 (reference)	0 (reference)
	30-34	0.08 (-0.04 to 0.21)	0.47 (0.34 to 0.59)	0.58 (0.42 to 0.75)
	35-39	0.20 (0.00 to 0.40)	0.90 (0.69 to 1.10)	1.18 (0.91 to 1.46)
	40 and above	-0.16 (-0.61 to 0.29)	0.74 (0.29 to 1.19)	0.58 (0.42 to 0.75)

The association between parental ages versus BPP cognitive score at conscription), when adjustment variables are added one at a time (n = 169,009, restricting to singletons born week 37-41 and birth weight between 3 and 4 kg n=90,687).

## Discussion

We found that while the offspring of both teen fathers and fathers above 35 years have impairment in cognitive ability in an unadjusted analysis, only the association between younger paternal age and impairment persisted after adjusting for maternal age, parental education and birth order. We found that older maternal age were associated with higher general cognitive scores in the offspring in both the unadjusted and adjusted results, though most clear when paternal age is adjusted for. In addition, our study clearly demonstrated how variables such as birth order, parental education, gestational age and birth weight influence offspring general cognitive scores.

The initial model (which adjusts parental ages mutually and for year of testing), suggested that the offspring of younger and older fathers did worse on the general cognitive measures – a finding consistent with the Israeli and the Swedish conscript studies [16,20]. The finding of no impairment in offspring of older fathers in the adjusted model is consistent with the most recent studies [18-20]. In contrast, the negative impact of being the offspring of a teenage father persisted in the full model, and is consistent with the findings from the Israeli conscript study [16], the Swedish school performance study [19] and the Swedish conscript [20].

The finding that older maternal age was associated with higher general cognitive scores for offspring is consistent with other studies adjusting for paternal age [17,18], whereas the relationship between maternal age and general cognitive scores has an inverted U-shape when paternal age is not adjusted for [16,20]. Maternal and paternal ages are strongly correlated (but one parent’s age does not ‘cause’ the age of the other parent to change) therefore, to separate their influence on IQ they should be mutually adjusted. Both unadjusted and adjusted estimates indicate that having more older siblings (i.e. being later in a sibship), and having a mother or father with less education were associated with lower general cognitive scores. Family size and educational outcomes are often socially patterned along socio-economic gradients (e.g. less well educated parents may have larger families) [23]. Thus, these factors may contribute to the finding that younger siblings in larger families (i.e. who tend to be the offspring of older fathers) have poorer general cognitive ability (as detected in the initial models). When these factors were included, the negative influence of having an older father did not persist.

For all the variables included in our analyses we find an association to general cognitive score. As expected, the largest differences were associated with maternal and paternal education. For paternal and maternal age, in the adjusted models, the mean difference in BPP scores between the age categories were smaller (e.g. less than 2 in the comparisons for maternal age, less than 1 in the comparisons for paternal age). At the individual level, these differences could potentially impact in subtle ways on educational outcomes. However, from the public health perspective, these otherwise small differences could potentially translate to a shift in educational and clinical outcomes that are more important at a population level [39].

The Swedish conscript study compared brothers to adjust for shared confounders [20] (e.g. parental education), however comparison of brothers may introduce confounding from non-shared confounder [40]. For some predictors of IQ it may be uncertain whether they are on the causal pathway from parental age to IQ, e.g. birth weight may be an effect of maternal age and birth order and therefore a mediator from maternal age and IQ. Whereas birth weight may not be a mediator between paternal age and IQ, since the main reason for paternal age and birth weight to correlate could be through maternal age (and birth order) [41]. The age of one parent does not ‘cause’ the age of the other parent, thereby the relation between paternal age (through maternal age) and birth weight is not ‘causal’, therefore to separate effects of paternal age and birth weight they should be mutually adjusted. The causal effect of paternal age on cognitive function in the offspring may be due to a selection into late fatherhood, which influence manifest when the child attends school, hence determination of the timing of the two variables is ambiguous. On the other hand if the reason for paternal age affecting cognition in the offspring is germ cell mutations, this effect can potentially go through birth weight, whereby birth weight is a mediating factors, which should not be adjusted for. The variables multiple pregnancy status, gestational age and birth weight were all associated with IQ, and they may be confounders or mediators, however, the associations between parental ages and IQ reach similar conclusions, whether these are adjusted for or not.

Our findings raise additional questions. In light of the known psychosocial benefits of having an older parent (e.g. more likely to have a planned pregnancy, more likely to have parents with better education and socio-economic status, more likely to have nurturing, supportive and stable home environments) [42,43], why aren’t the offspring of older parents (e.g. 40 or more years) doing substantially better on the general cognitive score? In the full model, the difference in BPP score between the offspring of older versus reference category mothers is around two unit points – a small difference at the individual level (20% of the standard deviation of this measure). In contrast, the impact of long maternal and paternal education on the measure of general cognitive ability is between 5 and 6 units in full model. A previous study based on infant and childhood cognitive measures [17] reported clear benefits in the offspring of older mothers, versus a steady worsening in the outcomes in the offspring of older fathers. It is feasible that, during the first years of life, the psychosocially-mediated factors are not sufficient to overcome any biologically-mediated factors (e.g. perhaps related to paternally-derived de novo mutations). We speculate that by young adulthood (e.g. the time of conscription), any potentially detrimental effects of paternal age may be overridden by potentially beneficial psychosocially-mediated factors. These factors could include age-related improvements in socioeconomic status, as well as more subtle gene-environment correlations. For example, parents with higher intelligence are not only more likely to go to university and delay parenthood, they are also more likely to enrich their children’s early life learning experiences (e.g. more books at home, more engagement at school). Combinations of these factors would tend to ‘ratchet’ up cognitive and educational outcomes in the offspring of older parents as they age and are exposed to potentially “enriched” environments.

The study has limitations related to the representative nature of the sample. Those with certain health conditions are exempt from conscription, thus individuals with some disorders (such as intellectual disability) were under-represented in our sample. Our sample consisted of males only. In addition, we only had one composite measure of general cognitive ability measured at one time point, and we had no information on the parents’ cognitive ability. Despite these factors, our study is based on a large sample, and was able to adjust for potential confounding variables, including parental education. While the impact of advanced paternal age has attracted considerable attention in recent years, by exploring models that incorporate important covariates, our study draws attention to potential adverse cognitive outcomes in the offspring of younger parents.
